# Supplementary material for: Tomography of memory engrams in self-organizing nanowire connectomes
Source: Nat Commun. 2023 Sep 27;14:5723. doi: 10.1038/s41467-023-40939-x (PMC10533552; doi:10.1038/s41467-023-40939-x)
Supplement: Supplementary file 1 — Supplementary Information [file 41467_2023_40939_MOESM1_ESM.pdf]

Tomography of memory engrams in self-organizing nanowire connectomes – Supplementary Information

Gianluca Milano[1*], Alessandro Cultrera[2], Luca Boarino[1], Luca Callegaro[2], Carlo Ricciardi[3*]

[1]Advanced Materials Metrology and Life Sciences Division, INRiM (Istituto Nazionale di Ricerca Metrologica), Strada delle Cacce 91, 10135 Torino, Italy.

[2]Quantum Metrology and Nanotechnologies Division, INRiM (Istituto Nazionale di Ricerca Metrologica), Strada delle Cacce 91, 10135 Torino, Italy.

[3]Department of Applied Science and Technology, Politecnico di Torino, C.so Duca degli Abruzzi 24, 10129 Torino, Italy.

*e-mails: g.milano@inrim.it; carlo.ricciardi@polito.it;

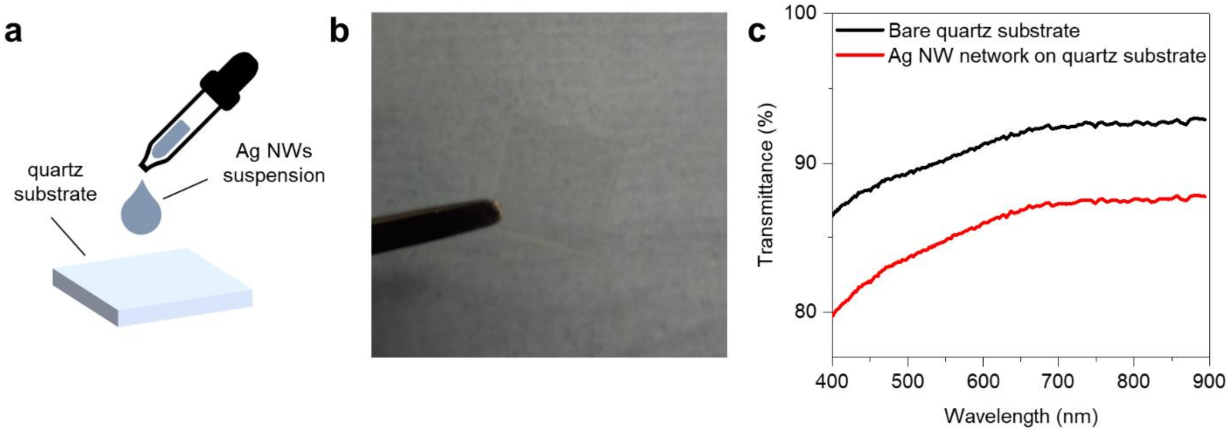

Supplementary Figure S1 | Ag NW network on a quartz substrate. a. Schematic representation of the fabrication of Ag NW networks where Ag NWs in suspensions are deposited by drop-casting on a 10 × 10 mm^2 quartz substrate. **b.** Photograph of the transparent Ag NW network deposited on the quartz substrate. **c.** Transmittance spectra of the Ag NW network on the quartz substrate compared to the transmittance spectra of the bare quartz substrate, showing the high transparency of the Ag NW network over a wide range of wavelengths.

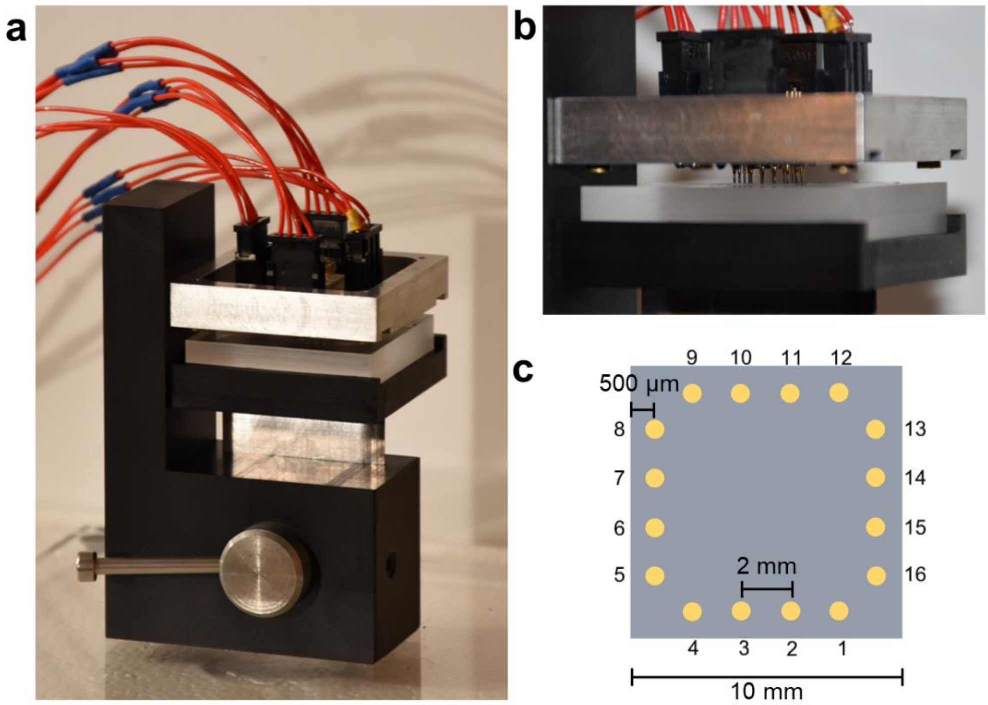

Supplementary Figure S2 | Experimental setup for multiterminal characterization. a. Photograph of the contact fixture for multiterminal electrical characterization. The sample, loaded on a milled plastic support, is placed in contact with spring-loaded electrode terminals by means of an actuator lever. **b.** Side-view image showing a detail of the 16 spring-loaded needle contacts (diameter of 40 μm, applied force limited to 0.15 N) that are placed in contact with the sample at 500 μm from its edges. **c.** Schematization of the position of needle probes contacting the NW network deposited on the quartz substrate.

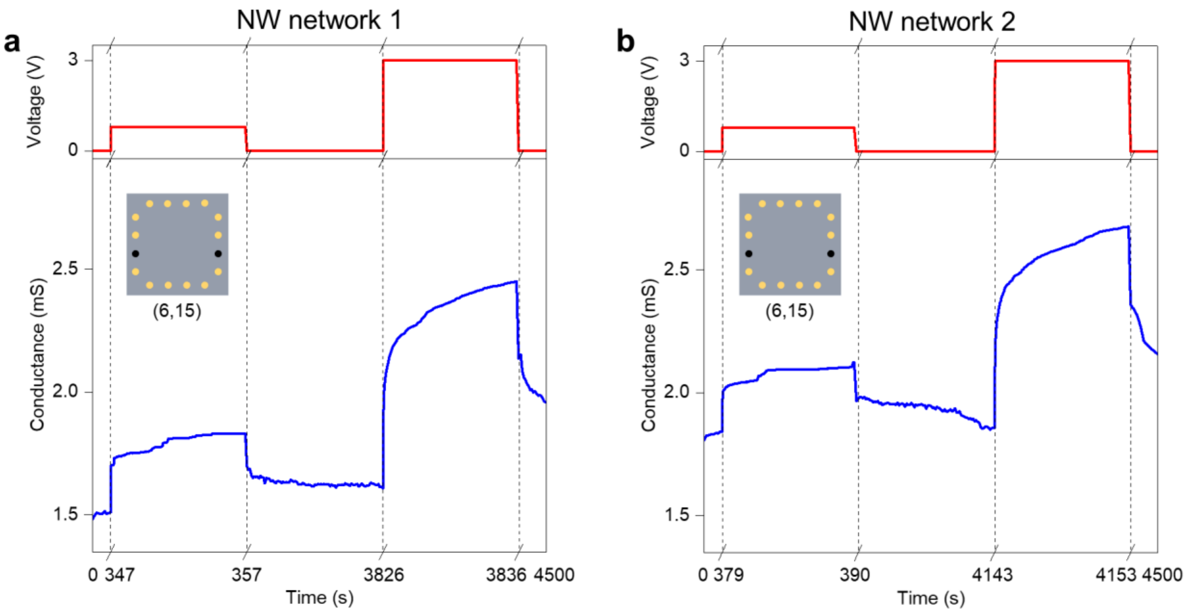

Supplementary Figure S3 | Reproducibility of the output under the same input in different NW networks. Repeatability of NW network dynamics in different, but with the same nominal characteristics, NW networks. Response of **a.** NW network 1 and **b.** NW network 2 of an arbitrary stimulation voltage waveform. Stimulation voltage waveforms, composed of two spaced voltage pulses with amplitude of 0.8 V and 3 V, respectively and length of 10 s, were applied to selected contacts of two NW networks with nominally identical characteristics. Contacts selected for stimulation (6 and 15) are highlighted in insets. The effective conductance in between pulses was monitored by applying a read voltage of 10 mV. Despite small variabilities in conductance values, it can be observed that the two NW networks show qualitatively similar dynamics, characterized by potentiation during voltage stimulation followed by spontaneous relaxation after stimulation.

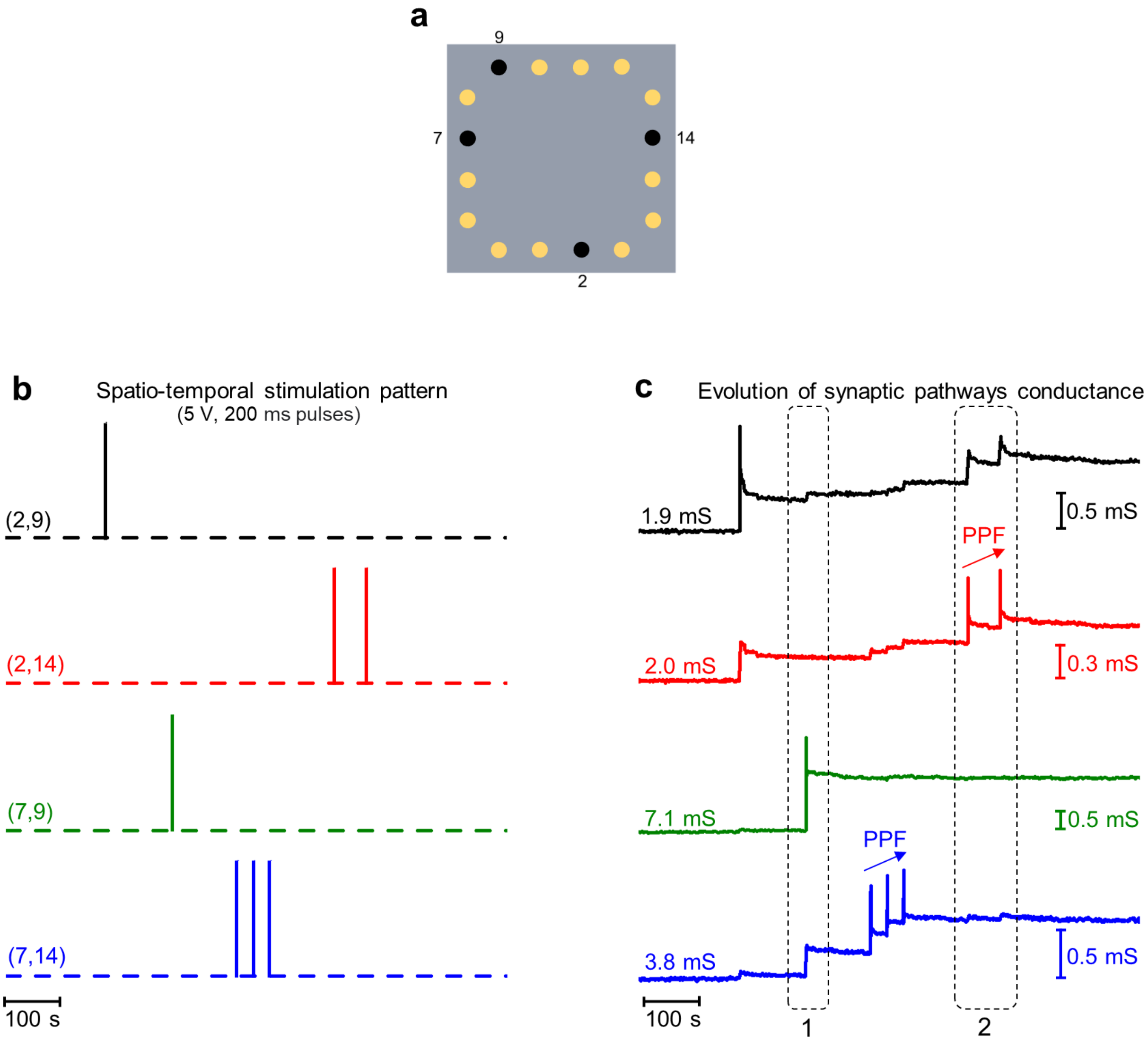

Supplementary Figure S4 | Influence of spatial location of neuron terminals on heterosynaptic effect. a. Schematic representation of the multiterminal configuration with highlighted terminals considered for the spatio-temporal stimulation pattern reported in Figure 2d and e. **b.** Spatio-temporal stimulation pattern and **c.** corresponding evolution of the conductance of synaptic pathways (from Figure 2d and e), where two examples of heterosynaptic phenomena are highlighted in circled areas. The circled area 1 in panel c refers to the temporal sequence where the synaptic pathway connecting terminals 7 and 9 is directly stimulated. As can be observed, besides changes in the effective conductance of the directly stimulated pathway (7,9), significant changes in the effective conductance were observed also in pathways (7,14) and (2,9), while almost no changes were observed in pathway (2,14). This behavior is related to the spatial location of neuron terminals. Indeed, while pathways (7,14) and (2,9) share neuron terminals with the directly stimulated (7,9) pathway, the pathway (2,14)

involves peripheral areas of the network with respect to the directly stimulated area. Similarly, in the circled area 2 where the pathway (2,14) is directly stimulated with paired pulses, negligible changes in the peripheral synaptic pathway (7,9) can be observed, while larger changes are observed in pathways (2,9) and (7,14). These examples show the dependence of heterosynaptic plasticity effects on the spatial location of neuron terminals due to the functional connectivity of the memristive network.

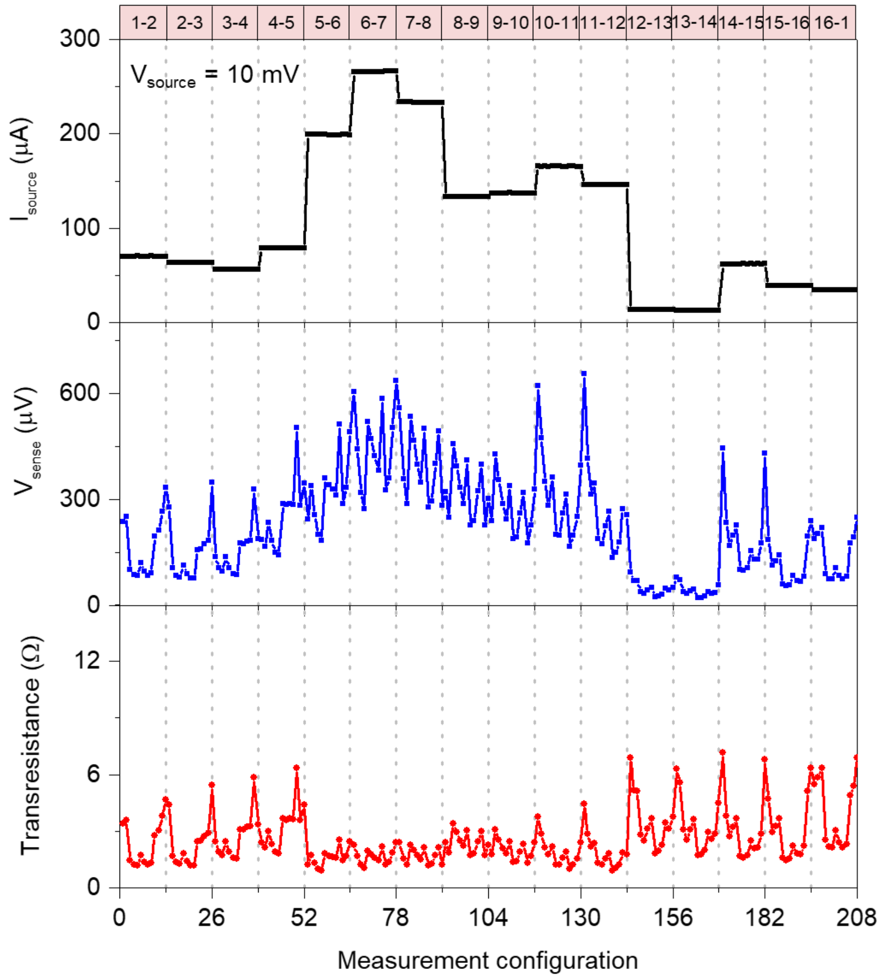

Supplementary Figure S5 | Experimental transresistance pattern from adjacent pattern measurement scheme. Source current (I_{source}) pattern, corresponding sense voltage (V_{sense}) pattern and transresistance pattern. Measurements were performed by applying a voltage of 10 mV to adjacent source terminals and measuring the corresponding I_{source}, while measuring V_{sense} across other pairs of adjacent terminals. The source configuration is labelled at the top.

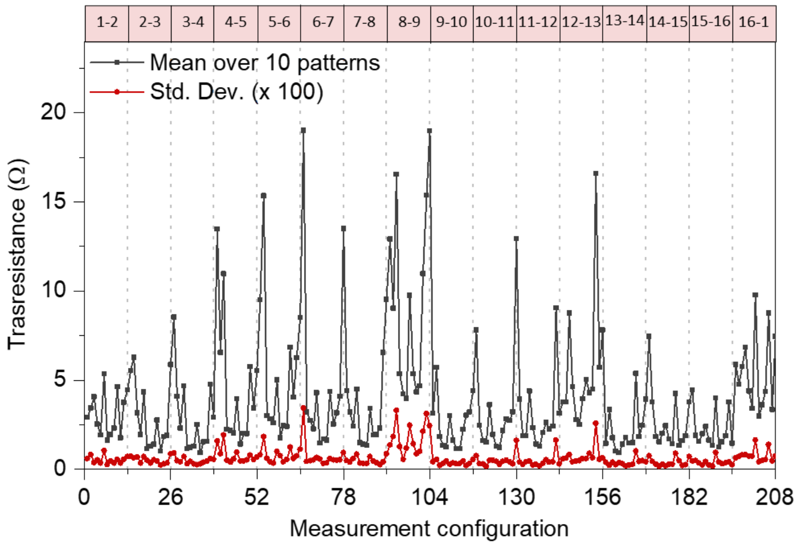

Supplementary Figure S6 | Reproducibility of the ERT measurement pattern. Mean and standard deviation (× 100) of measured ERT patterns evaluated by repeating the ERT measurement protocol 10 times on a NW network in the pristine state. A standard deviation in transresistance values < 0.5 % was observed, showing that the measurement protocol maximizes the signal-to-noise ratio while preventing the onset of sample alterations.

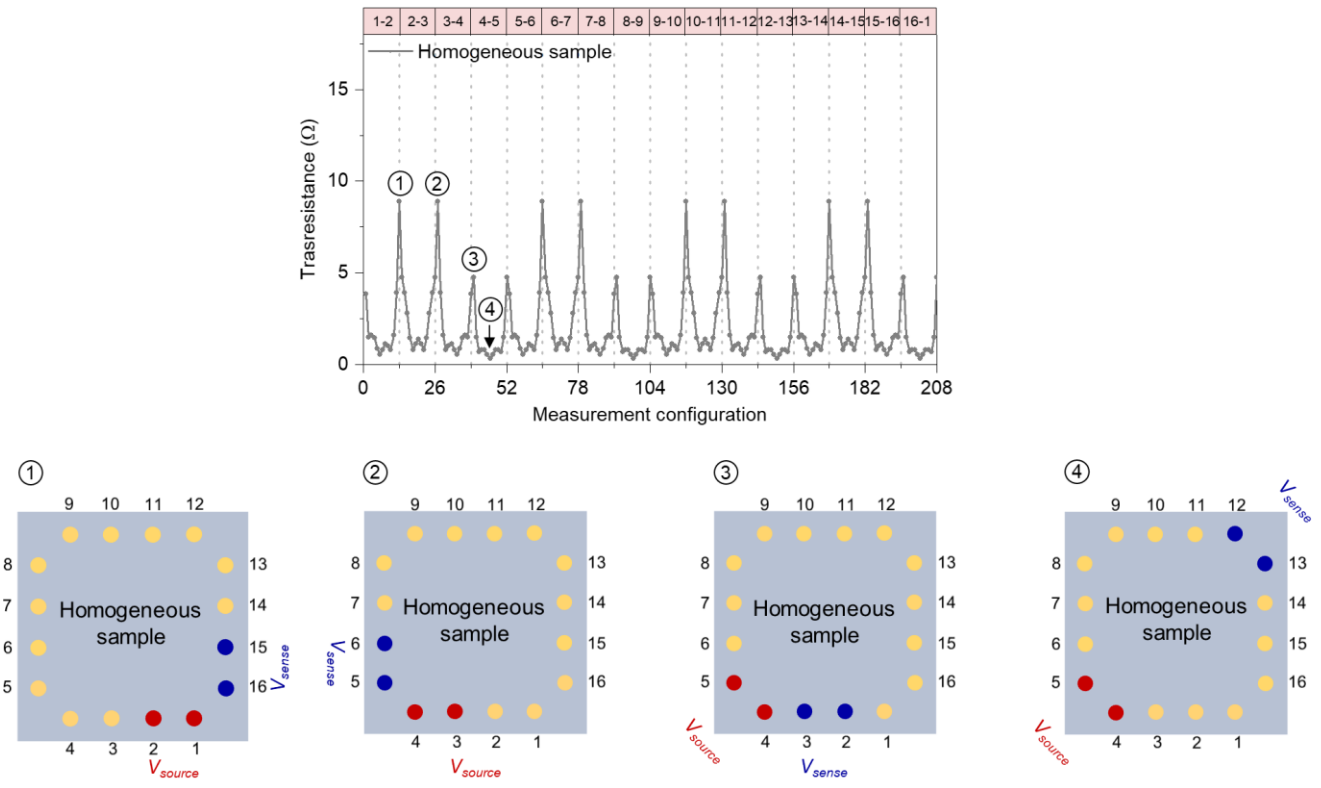

Supplementary Figure S7 | Transresistance pattern of a homogeneous sample (simulated). Transresistance pattern obtained with the adjacent protocol scheme of a simulated homogeneous sample with a median conductivity of 15.7 mS. Selected source-sense configurations corresponding to numbered measurements of the transresistance pattern are reported. In case of a homogeneous sample, geometrically symmetric source-sense configurations resulted in the same value of transresistance (refer to configurations 1 and 2). Configurations 3 and 4 correspond to sourcing a pair of contacts in the corner while sensing in adjacent and opposite pairs of contacts, respectively.

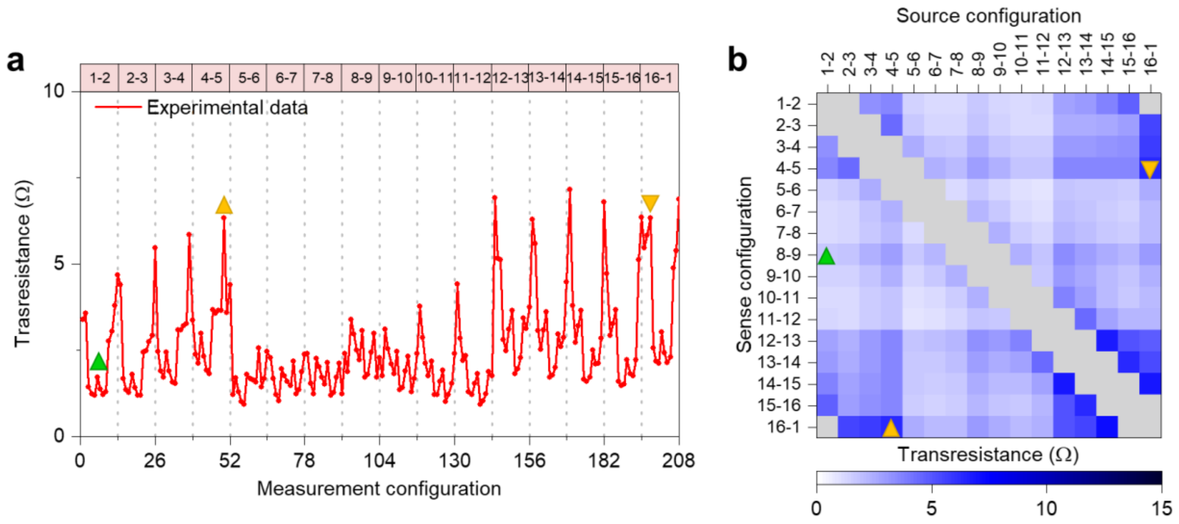

Supplementary Figure S8 | Reconstruction of the impedance matrix from the transresistance pattern. a. Experimental transresistance pattern of an Ag NW network and **b.** corresponding impedance matrix reconstructed from transresistance data. Matrix elements highlighted with triangle markers in panel b correspond to measurements marked in panel a. Orange triangles mark two symmetric configurations. The ERT-wired sample can be considered a common loop multi terminal network[1], where the transresistance measurements obtained on the sample are the entries of the impedance matrix Z associated with the electrical network.

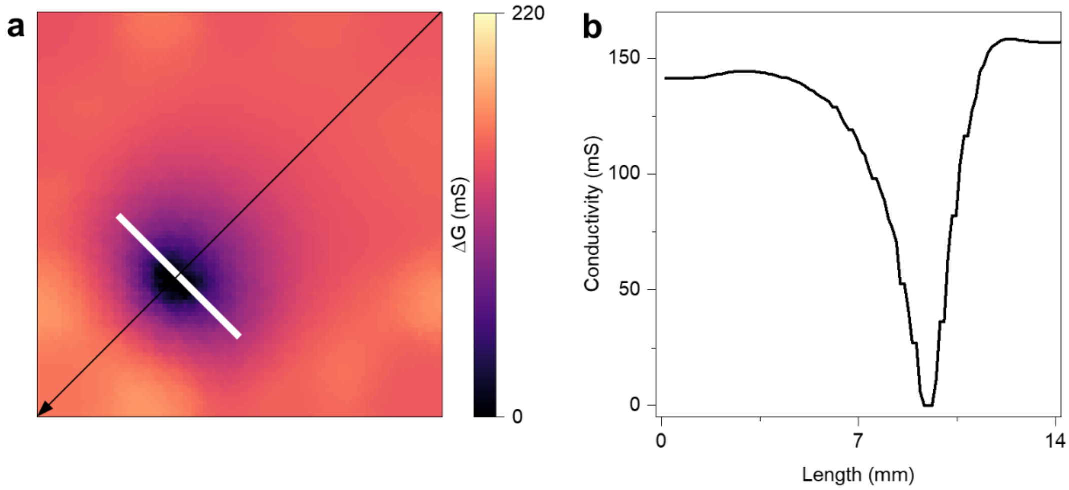

Supplementary Figure S9. Experimental investigation of the spatial resolution of the ERT setup. a. Fluorinated tin oxide (FTO) thin film with a thin linear cut (white marker of about 50 μm). Before the linear cut, the FTO sample was characterized by a uniform conductivity of 150 mS [2]. **b.** Conductivity dip observed in the diagonal conductivity profile of the sample (in the direction of the black arrow in the map of panel a), where the full width at half maximum corresponds to ≈ 1.7 mm. Details on ERT spatial resolution in Supplementary Note 5.

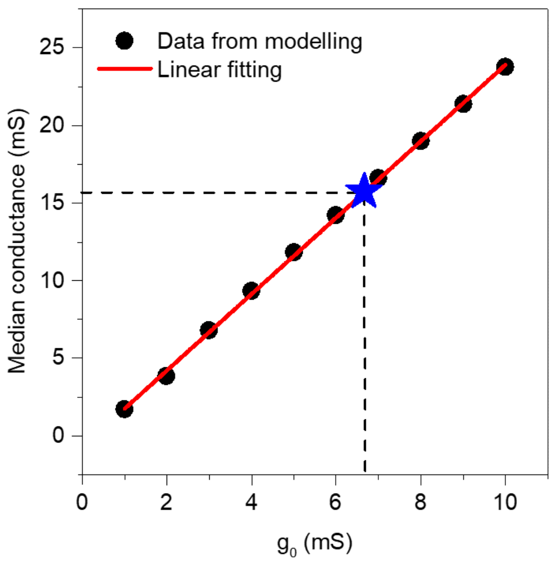

Supplementary Figure S10 | Extraction of grid-graph model parameters in the pristine state. The value of the pristine conductance of each grid-graph edge g_0 is estimated by interpolating the median conductance of the ERT map resulting from the grid-graph model and the median conductance of the experimental map reported in Figure 2d. In the pristine state, each edge of the grid-graph was assigned to the same g_0 value under the assumption of a homogeneous NW network. As a first step, the grid-graph model was initialized by imposing a g_0 value of conductance to each edge. Then, the transresistance pattern and the corresponding impedance matrix were acquired to obtain the conductivity map of the grid-graph model by means of ERT reconstruction. Subsequently, the median conductance of the pixels of the map was calculated. The same procedure was repeated for different values of g_0, unveiling a linear relationship in between the median conductivity of the reconstructed map and the g_0 edge conductance. By exploiting a linear interpolation of data, the g_0 value of edges was extrapolated such that the median conductance of the simulated conductivity map matches well with the conductivity map reconstructed from experimental data as reported in Figure 2.

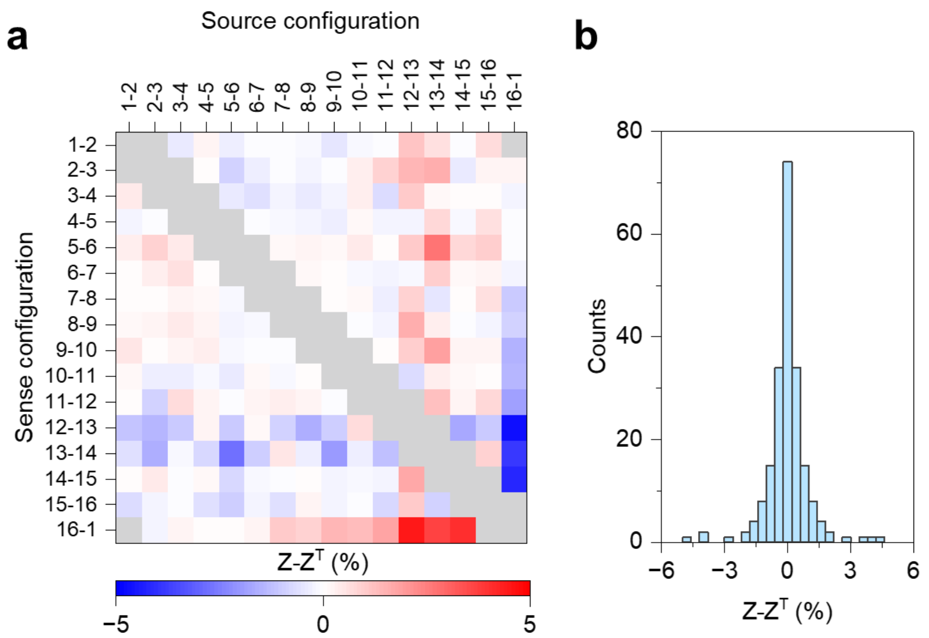

Supplementary Figure S11 | Reciprocity of the NW network and symmetry of impedance matrices. a. Difference matrix resulting from the difference (in percentage) in between the impedance matrix Z and its transpose Z^T exploited to evaluate the symmetry of Z (details in Supplementary Note 6) and **b.** corresponding histogram of the difference matrix elements. The $Z - Z^T$ matrix and histogram have been reconstructed from the experimental impedance matrix of the NW network reported in Figure 2d. The reciprocity of the network is reflected in a symmetric matrix with values of the $Z - Z^T$ matrix < 5%, where low deviations from a perfect symmetry can be attributed to measurement noise.

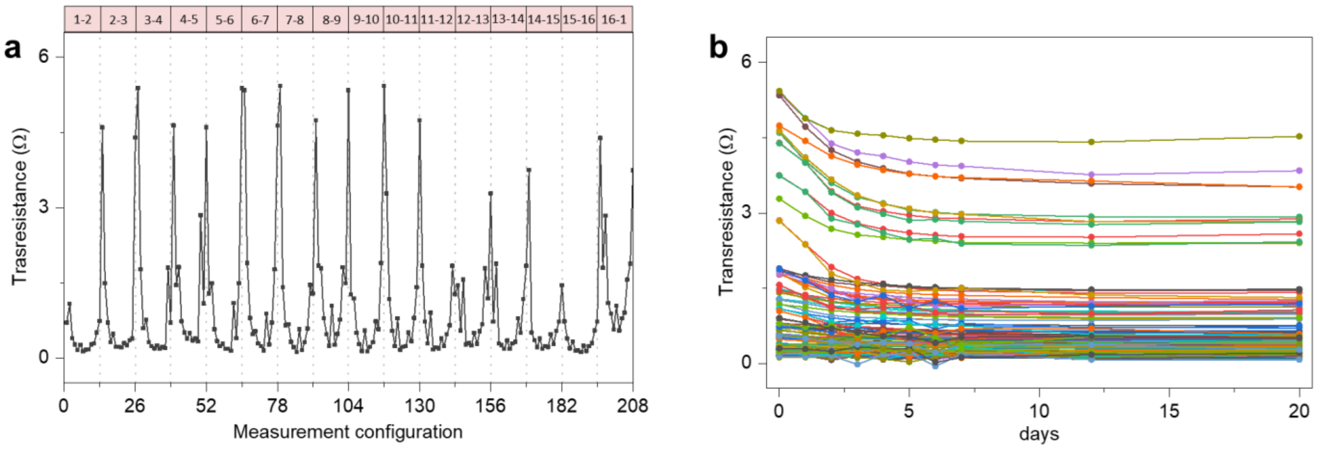

Supplementary Figure S12 | Long-term stability of NW networks. a. Transresistance pattern of an Ag NW network after deposition and **b.** evolution over time of transresistance values of all the 208 configurations, monitored by placing the ERT setup in a hermetically closed box to limit the interaction with the environment. After initial stabilization, transresistance values of the multiterminal NW network tend to stabilize to near constant values and no network failures were observed after 20 days.

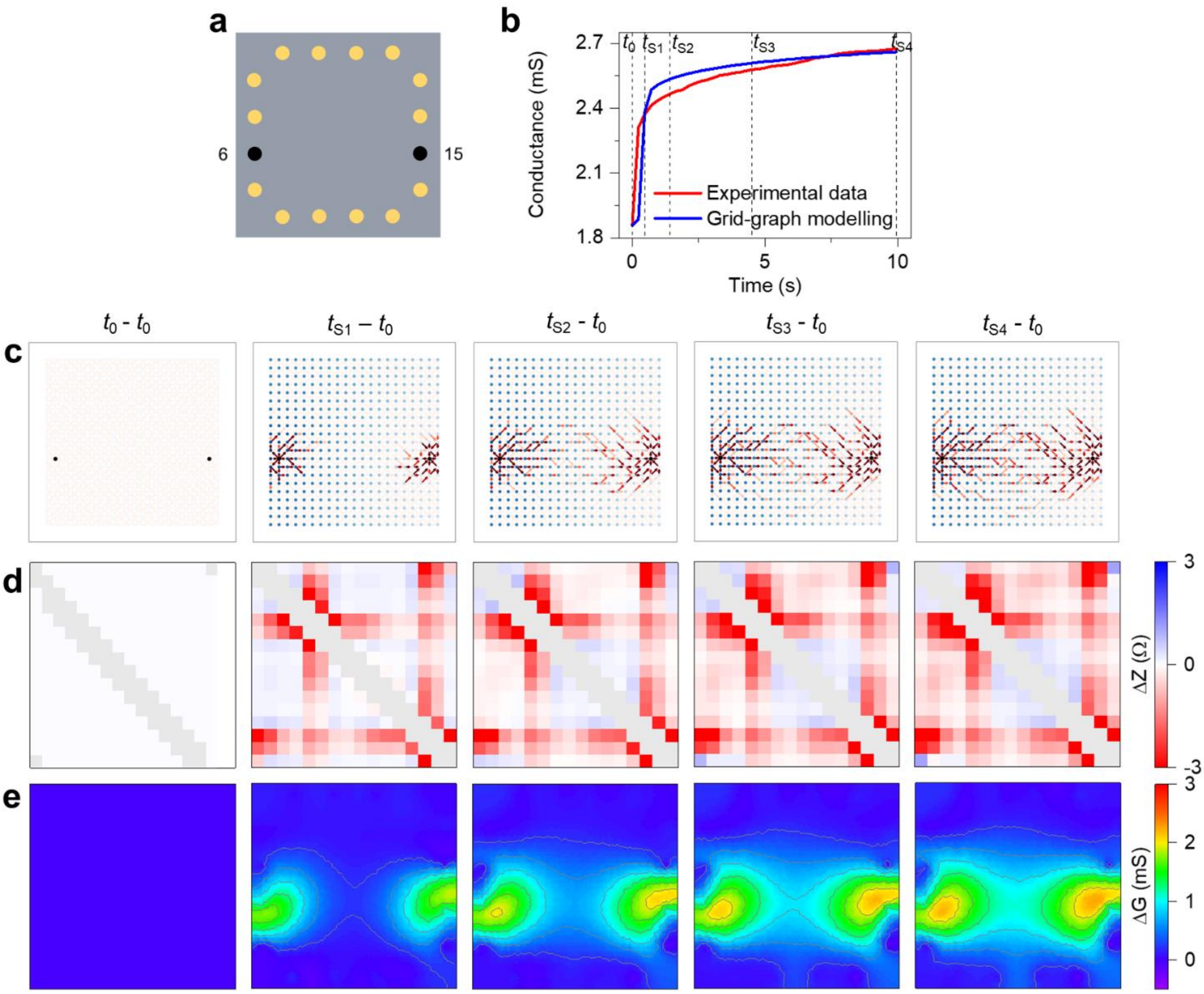

Supplementary Figure S13 | Mapping dynamic formation of synaptic pathways across the NW network connectome. a. Schematic representation of the multiterminal configuration with highlighted terminals of the directly stimulated synaptic pathway and **b.** experimental and simulated evolution of the effective conductance in between the selected pair of contacts during potentiation (detail of Figure 3b). **c.** Evolution of the memristive network by grid-graph modelling (red intensity is proportional to the edge conductance, blue intensity is proportional to the node voltage), **d.** corresponding simulated differential impedance matrices and **e.** corresponding simulated differential conductivity maps by ERT reconstruction during stimulation (Supplementary Movie 2). The grid-graph model and the reconstructed maps enable direct investigation of the synaptic potentiation

dynamics at the macroscale, showing the gradual formation of a potentiated conductive pathway growing over time starting from the stimulated contacts along the electric potential gradient.

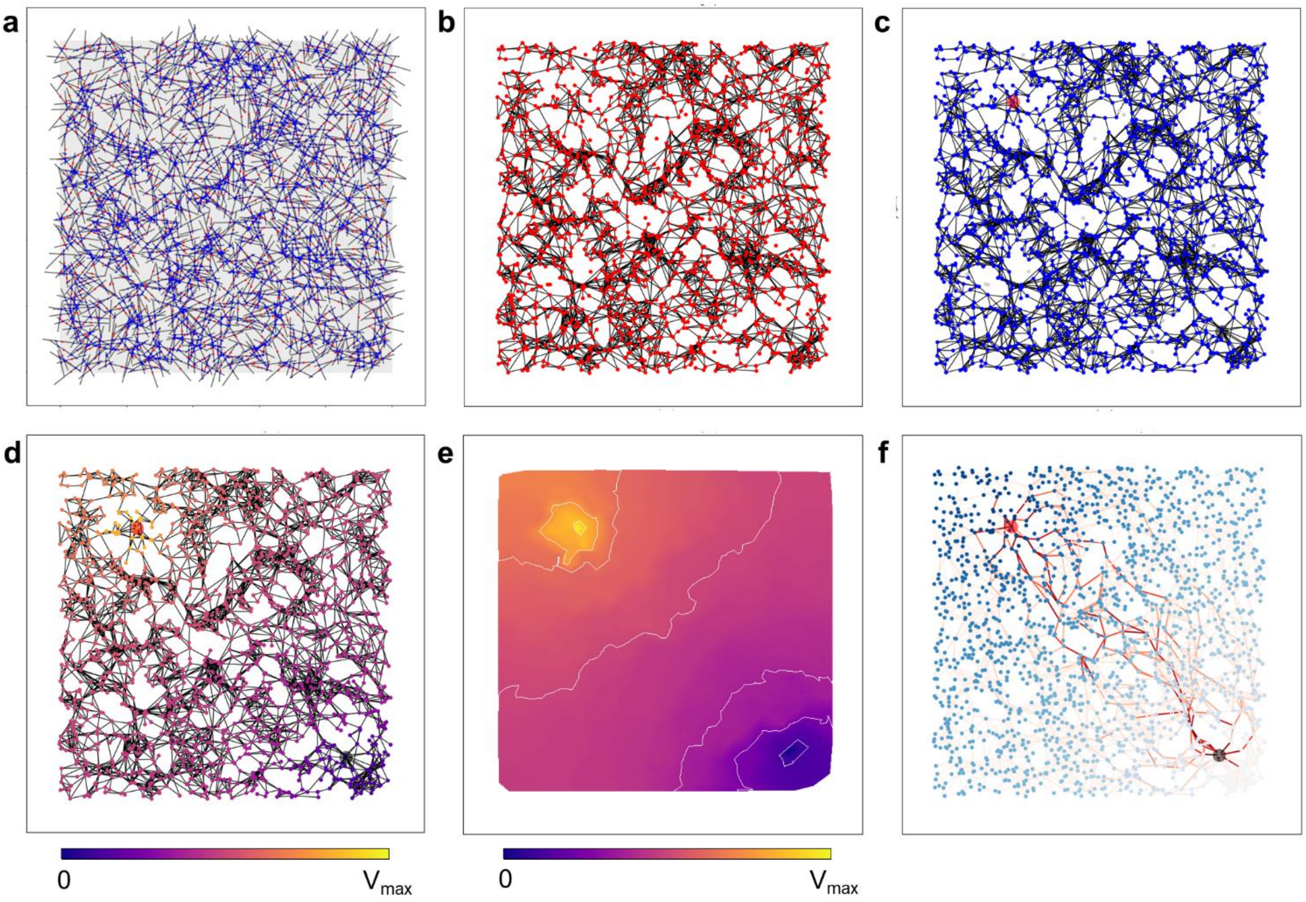

Supplementary Figure S14 | Emergent behavior of the NW network connectome at the micro/nanoscale. a. NW network topology simulated by dispersing 1D objects (1800 NWs) on a 2D plane (500 × 500 μm^2), where red dots represent NW midpoints while blue dots represent NW junctions, and **b.** corresponding graph representation. **c.** Electrical backbone of the network when stimulated in between source (red marked node, upper left) and ground node (black marked node, bottom right). **d.** Corresponding visualization of the potential distribution across graph nodes when a voltage difference is applied between these nodes. **e.** Corresponding voltage distribution across the 2D plane. **f.** Activation pattern of the network after stimulation in between source and ground nodes with a voltage pulse, showing the emergence of a conductive pathway composed of multiple branches that connects source and ground nodes. Red intensity is proportional to edge conductance, blue intensity is proportional to node voltage.

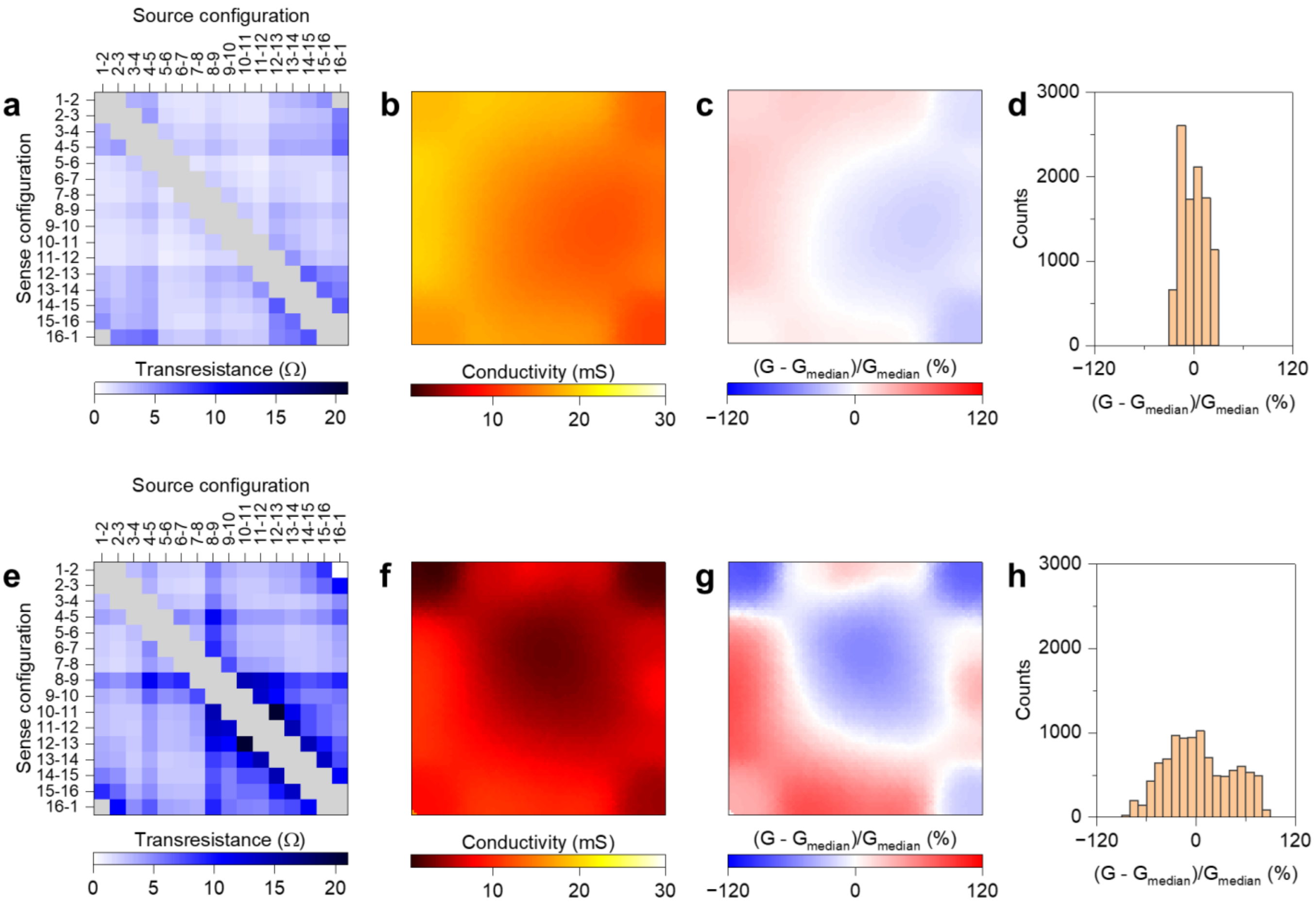

Supplementary Figure S15 | Comparison of the homogeneous and the non-homogeneous network. a. Impedance matrix and **b.** conductivity map of the high density nearly homogeneous NW network, **c.** map of the percentage variation of conductivity across the network with respect to the median conductance value of the network (median conductance of 15,8 mS) and **d.** corresponding pixel histogram of conductance variations. **e.** Impedance matrix and **f.** conductivity map of the non-homogeneous NW network, **g.** map of the percentage variation of conductivity across the network with respect to the median conductance value of the entire network (median conductance of 5,9 mS) and **h.** corresponding pixel histogram of conductance variations. A comparison of the impedance matrices shows a less uniform impedance matrix in case of the non-homogeneous network compared to the homogeneous sample that reflects in a less homogeneous spatial distribution of conductivity across the network. The non-homogeneous network, that is characterized by a lower median conductance, is characterized by larger percentage variations of conductivity over the network.

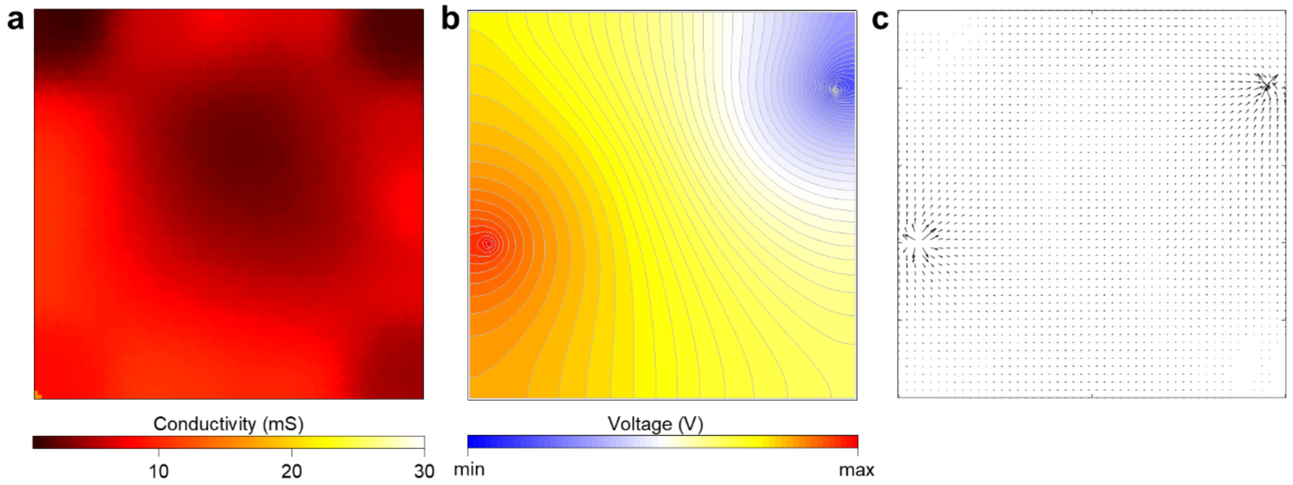

Supplementary Figure S16 | Electric potential and current distribution in the non-homogeneous connectome. (a) Experimental conductivity map of the non-homogenous NW network reported in Figure 4a and (b) corresponding distribution of the electric potential over the pristine conductive map when a potential difference is externally applied in between terminals 6 and 13. It can be noticed that the higher pristine state conductivity near terminal 6 (terminal on the left) results in a large nearly equipotential area surrounding this electrode that acts as a nearly equipotential virtual electrode and drives the topology-dependent formation of the conductive pathway. (c) Map of current vectors across the pristine state corresponding to the electrical potential distribution reported in panel b. In this case, the effect of the nearly equipotential area near terminal 6 can be observed by the wider spread of current lines around this area. In panel c, the arrow length is proportional to current density.

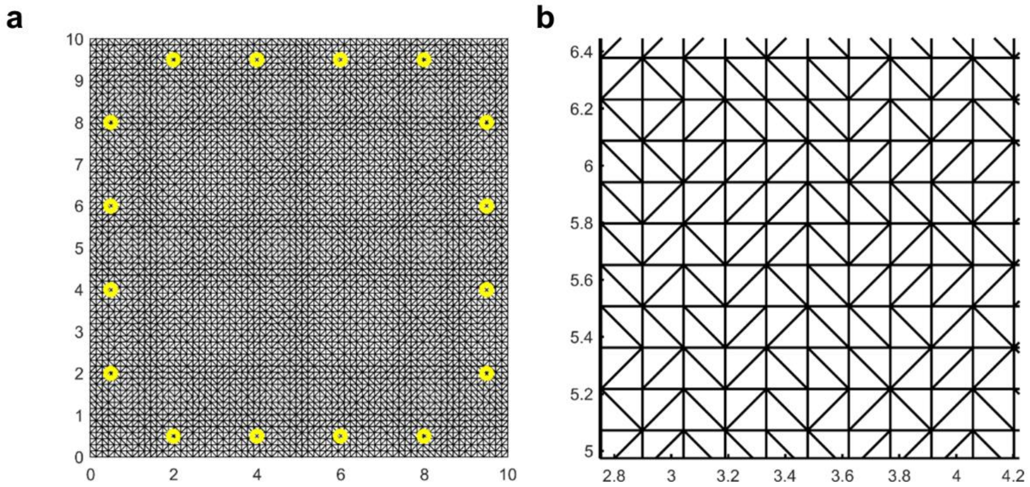

Supplementary Figure S17 | Finite element mesh model for ERT map reconstruction. a. Finite element mesh model used in EIDORS for image reconstruction and **b.** a detailed view of the mesh model. The model implements 9554 triangular elements and the 16 contacts corresponding to the experimental fixture. The contacts are modeled as point-contacts at single mesh nodes. Lateral dimensions coordinates are expressed in mm.

Supplementary Table 1. Modelling parameters. Parameters of the potentiation-depression balance-rate equation exploited for regulating the edge dynamics of the grid-graph model (refer to Supplementary Note 4), extracted from interpolation of the two-terminal experimental characteristics of potentiation/relaxation reported in Figure 3b.

Parameters	Value
$G_{\min}$	2,1 mS
$G_{\max}$	3,6 mS
η_P	102 V^{-1}
η_D	30 V^{-1}
κ_{P0}	$5 \cdot 10^{-6}$ μs^{-1}
κ_{D0}	$1.8 \cdot 10^{-3}$ s^{-1}

Supplementary Table 2. Comparison of ERT with other characterization techniques for direct visualization of conductive pathways in self-organizing nanonetworks.

Technique	Measurand	Resolution (Pixel size)	Scanning area	Acquisition time
**ERT (this work)**	Local conductivity	≈ 2 mm	≈ 1 × 1 cm^2	≈ 40 s (non-scanning technique)
**Lock-in thermography** (ref.[3])	Infrared emission (Current is dissipated within current transmitting pathways, IR intensity can be then converted to temperature)	3 µm (but the spatial resolution is several times worse that the pixel size because of the optical limit of the lens)	≈ 1 × 1 mm^2	50 s (non-scanning technique)
**Scanning Electron Microscopy** (ref.[4,5])	Secondary electrons (Passive voltage contrast images, produced by a low energy electron beam of 2-4 kV)	≈ 10 nm*	up to ≈ 100 × 100 μm^2	ms – s* (scanning technique)▲
**Conductive-AFM** (ref.[5])	Local current (Current flowing in between the C-AFM tip and a reference electrode)	≈ few nm*	≈ 50× 50 μm^2	≈ min* (scanning technique)▲

* Not specified, estimation based on technique properties.

▲ Acquisition time scales quadratically with the scanning area in scanning techniques

Supplementary Table 3. Comparison of the resolution of mapping techniques and synaptic densities in NW networks and human brain.

NW network (2D)<br>synaptic junction density: ≈ $2 \cdot 10^6 – 3 \cdot 10^6$ mm^{-2}		
**Technique**	**Pixel size**	**Synaptic junctions/pixel**
ERT (this work)	≈ 2 mm	≈ $8 \cdot 10^6 – 1.2 \cdot 10^7$
**Human brain (3D)**<br>synaptic density: ≈$11 \cdot 10^8$ mm^{-3} * (ref. [6])		
**Technique**	**Pixel size**	**Synaptic junctions/pixel**
fMRI	≈ 3 - 4 mm<br>pixel size of 500 um or less may be achieved with higher field magnets (7 T) (ref.[7])	≈ $3 \cdot 10^{10}$ - $7 \cdot 10^{10}$
PET	≈ 5 - 10 mm<br>limited by the size of the gamma-ray detectors as well as the positron-electron annihilation range (ref.[7])	≈ $1 \cdot 10^{11}$ - $1 \cdot 10^{12}$
EEG	≈ 10 - 20 mm<br>limited by the fact that unique reproduction of dipoles is not possible from scalp-based measurements and regularization should be employed for model estimation (ref.[7])	≈ $1 \cdot 10^{12}$ - $9 \cdot 10^{12}$

*Average number of synaptic density in adult life

Supplementary Note 1 | Estimation of NW density and NW junction density.

The NW density estimation was performed by knowing the NW network areal mass density of fabricated NW networks (in the range ≈ 99 – 136 mg m^{-2}), calculated by knowing the weight percentage of NWs in solution, the solution volume deposited on the substrate, the average NW dimensions, and the substrate surface area.

Estimated NW density: $n \approx 5.7 \cdot 10^4 - 7.8 \cdot 10^4$ mm^{-2}

The estimation of NW junction density was performed through the formula $j = \frac{1}{2}P \cdot \pi n D = \frac{1}{2}P\pi n^2 L^2$ [8], where P = 0.2027 is the contact probability of 1D objects on a 2D plane (irrespective of the network density) [9], n is the NW density, D the normalized NW density and L the NW length. The calculated junction density is in line with previous estimation of ≈10^6 NW junctions mm^{-2} from SEM imaging performed in a previous work [10].

Estimated NW junction density: $j \approx 1.6 \cdot 10^6 - 3.1 \cdot 10^6$ mm^{-2}

Supplementary Note 2 | Resistive switching mechanism in NW networks.

The emergent memristive behavior of NW networks arises from resistive switching events occurring in network elements, as detailed in a previous work [10] where the resistive switching of single elements composing the network was analyzed by considering devices based on single NW cross-point junctions and single NWs. The network can experience *weight plasticity* related to the change of conductance of NW junctions depending on the history of experienced electrical stimulation. In this case, the switching mechanism relies on the formation/rupture of a metallic bridge connecting the two NW metallic cores at the intersection point driven by the electric field. This switching mechanism is typically volatile, meaning that the conductance state of the junction can spontaneously relax to the ground state after stimulation due to the spontaneous dissolution of the metallic conductive bridge. The spontaneous dissolution of the metallic bridge is related to energy minimization effects (details on the physical mechanisms driving the spontaneous dissolution of the filament can be found in refs.[11–13]). The timescale of the conductive bridge relaxation depends on the history of applied voltage and current. In addition, the network can experience *wiring plasticity* that can lead to electrically-induced changes in the network topology. This effect relies on the rupture and rewiring of NWs under electrical stimulation driven by Joule heating and electromigration-driven breakdown events. When electrically stimulated, the current across the network is distributed according to Kirchhoff's law and is regulated by the conductance of each network element. Resistive switching events in network elements cause a redistribution of currents and voltages across the network, inducing conductance changes also in other memristive elements through an avalanche effect. These effects give rise to the emergent memristive behavior of the system.

Supplementary Note 3 | ERT measurement protocol for mapping nanowire networks.

ERT has been demonstrated to be a reliable technique for electrical conductivity mapping of thin films - e.g., transparent conductive oxides[14], and 2D materials like graphene[2]. More recently, it was demonstrated that it is possible to map the conductivity of metallic nanowire networks as transparent conductive materials[15]. Here, it was shown that the ERT technique can be exploited to map the conductivity of NW networks with a wide range of areal mass densities (60 - 180 mg/m^2). Importantly, it was reported that the conventional ERT protocols are not suitable for mapping NW networks. Typically, ERT is performed using “current-controlled” measurement protocols, in which the source current I_{source} is kept constant. This means that for each of the contact configurations involved the applied voltage necessary to inject such a current is not under control (it depends on the resistance seen from the selected contacts). Larger voltages applied to pairs of contacts that exhibit larger resistances to inject I_{source} were observed to induce sample alterations when considering NW networks related to unwanted electromigration phenomena. To avoid this issue, a new measurement protocol was developed to perform non-invasive ERT mapping.[16] This implementation is based on a “voltage-controlled” measurement protocol. Differently from the conventional “current-controlled” protocol, the “voltage-controlled” protocol prescribes the source voltage (V_{source}) to be kept constant at a low value that do not induce switching events in the network while I_{source} can vary depending on the contact configuration. In this framework, this protocol makes it possible to perform non-invasive ERT measurements on one side preventing sample alterations and on the other side maximizing the signal to noise ratio in the transresistance measurements. A detailed description of the “voltage-controlled” measurement protocol can be found in our previous work.[16]

Supplementary Note 4 | Modelling the NW network as a grid-graph.

The grid-graph modelling approach here exploited to model the emergent behavior of the homogeneous and high-density NW network relies on two main steps [17]: *i)* approximation of the NW network as a continuous medium, *ii)* parcellation of the 2D domain and approximation as a regular grid graph.

i) Approximation of the NW network as a continuous medium

Theoretical and experimental investigation of NW networks by Forrò et al.[18] and Sannicolo et al.[19], respectively, revealed that the voltage distribution across a sufficiently dense NW network can be approximated to the voltage distribution observed in a continuous medium. By considering the NW network normalized density described by the equation $D = n_w L^2$, where n_w is the number of NWs per unit area while L is the NW length, the approximation holds already for $D > 2D_c$, where $D_c = 5.63$ is the percolation critical density.[18] This approximation holds for NW networks exploited in this work characterized by a normalized density of $D >\approx 91$, where D was estimated by considering an areal mass density (AMD) of ~ 99 mg m^{-2}, an average diameter of 115 nm and a length of 40 µm (see Methods). Moreover, it is worth noticing that the approximation of the NW network as a continuous medium is in accordance with resistance grid network models, where the effective resistance measured in between 3 or 4 lattice spacing apart was reported to well match with that of a continuous uniform sheet.[20]

ii) Parcellation of the 2D domain and approximation as a regular grid graph

The 2D plane representing the continuous material is parceled by means of a $N \times N$ mesh, and the pixeled network is represented through the grid-graph model built by associating a node to each pixel of the parceled domain and by introducing memristive edges to let neighbor nodes communicate (diagonal edges are introduced to make the network isotropic). A similar parceling of a continuous material was performed to model memristive materials and insulating layers of resistive switching

cells[21,22]. The consistency of this approach is supported also by the observation that the emergent memristive behavior of self-assembled networks can be described by an equation where complexity can be reabsorbed into the effective parameters of a single memristive element, as recently theoretically shown through a mean-field theory approach[23].

Previous results demonstrated that this modelling approach is able to emulate main features of the emergent memristive functionalities of NW networks and represent a versatile model for exploiting computing implementation strategies [17,24,25]

Supplementary Note 5 | Spatial resolution and traceability of ERT mapping.

Figures of merit for the quality of an ERT reconstruction have been proposed, mostly related to the use of ERT as an imaging technique, hence quantifying the image resolution, contrast, and geometrical accuracy.[26] These are dependent on the number of contacts, the measurement stimulation pattern, the measurement noise, the spatial position and shape of the object to be identified, and the specific reconstruction algorithm employed. In a previous work,[14] the analogue of the point spread function (PSF) of optical systems was considered to quantify the spatial resolution of ERT. Simulations suggested that one of the main limiting factors is the number of contacts when using a Gauss-Newton solver (the solver used in the present work). Hence, in first place, experimentally, for a comparable number of contacts (16 in this work), the spatial resolution can be considered of the order of contact distance (≈ 2 mm).

The spatial resolution of the 16-contacts ERT setup and reconstruction algorithm was experimentally investigated by considering a fluorinated tin oxide (FTO) thin film, initially of uniform conductivity of 150 mS ^{2}, that had been later damaged with a thin linear cut (about 50 μm). The experimental conductivity map obtained by ERT on the FTO sample with the linear cut is reported in Supplementary Figure 9a. The spatial resolution described by the full width at half maximum of the conductivity dip over the sample diagonal (Supplementary Figure 9b) was measured to be ≈ 1.7 mm. This value is comparable with the distance between two adjacent contacts of 2 mm (on each edge of the contacts array). Hence the effective-pixel lateral size of ≈ 2 mm can be considered reasonable in the ERT setup used in this work.

Traceability to the SI for ERT is linked to the calibration of the instrumentation but also to the validation of the reconstruction algorithms. The individual transresistance measurements are traceable to the SI through the periodic calibration of the electrical instruments involved; the uncertainty is better than 1%. In terms of conductivity maps, there is still very little work available in literature. It has been shown that for simple conductivity distributions, the reconstruction error due to

the measurement accuracy which is typical of laboratory grade instrumentation is small.[27] The assessment of the final ERT image accuracy is an open problem. The accuracy of the conductivity values given by the reconstructed maps has been validated, also by comparison with other measurement techniques [10], [11], on reference samples.

Supplementary Note 6 | Reciprocity of the NW network and symmetry of impedance matrices.

The same transresistance value is expected by sourcing a pair of selected contacts and sensing another selected pair of contacts or vice versa ($R_{i,j;k,l} = R_{k,l;i,j}$) since the NW network represents a reciprocal passive network where, according to the reciprocity theorem[28], the impedance matrix is symmetric $Z_{p,q} = Z_{q,p}$. The symmetry of the impedance matrix can be evaluated by considering the variations in between the impedance matrix Z and its transpose Z^T, as for example reported in Supplementary Figure S11.

Supplementary Note 7 | Tailoring stimulation for short-term synaptic plasticity effects.

The competing effects of memory enhancement and spontaneous decay related to the formation and spontaneous dissolution of conductive filaments in memristive cells drives the transition from short-term synaptic plasticity to long-term synaptic effects. In conventional memristive cells, the transition from a short-term memory regime to long-term memory regime can be controlled through the applied electrical stimulation (voltage/current amplitude, pulse length, pulse rate, etc.) by regulating the filament lifetime over several order of magnitude.[13,29] In case of NW networks, our previous works performed on nearly homogeneous networks showed that it is possible to identify appropriate stimulating conditions that drives mainly volatile switching events in network elements, making possible to operate the network in the short-term memory regime.[10,24] In this context, stimulation in Figure 3b was selected to obtain short-term plasticity effects while minimizing long-lasting changes.

Supplementary Note 8 | Grid-graph modelling NW network in the short-term memory regime.

Short-term memory of network elements was modeled by regulating memristive dynamics of graph edges with a potentiation-depression rate-balance equation[30] that allows to simulate the network response and emergent network behavior when stimulated with arbitrary input voltage waveforms, as detailed in our previous works.[24] In brief, the current I_{ij} flowing in the edge connecting the *ith* and *jth* nodes is given by the relation:

$$I_{ij} = \left[G_{\min}\left(1 - g_{ij}\right) + G_{\max} \cdot g_{ij}\right] \Delta V_{ij} \quad (1)$$

where g_{ij} is the normalized conductance (values in between 0 and 1), $G_{\min}$ and $G_{\max}$ are the minimum and maximum edge conductances, respectively, while ΔV_{ij} is the voltage difference between i^{th} and j^{th} node. The evolution over time of the edge conductance is represented by the state equation:

$$\frac{dg_{ij}}{dt} = \kappa_{P,ij}\left(V_{ij}\right) \cdot \left(1 - g_{ij}\right) - \kappa_{D,ij}\left(V_{ij}\right) \cdot g_{ij} \quad (2)$$

where $\kappa_{P,ij}\left(V_{ij}\right)$ and $\kappa_{D,ij}\left(V_{ij}\right)$ are the potentiation and depression rate coefficients that, as expected for diffusion of ions, are assumed to be function of the applied voltage through exponential relations:

$$\kappa_{P,ij}(V_{ij}) = \kappa_{P0} exp\left(+\eta_P V_{ij}\right) \quad (3)$$

$$\kappa_{D,ij}(V_{ij}) = \kappa_{D0} exp\left(-\eta_D V_{ij}\right) \quad (4)$$

where $\eta_{P,}\eta_D > 0$ are transition rates while $\kappa_{P0}, \kappa_{D0} > 0$ are constants. By assuming a simulation timestep $\Delta t > 0$, the recursive solution of equation (2) can be expressed as:

$$g_{ij,t} = \frac{\kappa_{P,ij}}{\kappa_{P,ij}+\kappa_{D,ij}} \left\{1 - \left[1 - \left(1 + \frac{\kappa_{D,ij}}{\kappa_{P,ij}}\right) g_{ij,t-1}\right] e^{-(\kappa_{P,ij}+\kappa_{D,ij})\Delta t}\right\} \quad (5)$$

where $g_{ij,t}$ and $g_{ij,t-1}$ are normalized conductances at times t and $t-1$ of the corresponding memristive edge ij. Eq. (5) is exploited to update the conductance of each graph edge at each timestep.

The electrical solution of the graph for each simulation timestep is performed by means of Modified Voltage Node Analysis (MVNA) algorithm (a generalization of the Voltage Node Analysis algorithm) that allows to evaluate the voltage distribution across graph nodes and, subsequently, currents across edges. Parameters of the rate-balance equation to regulate edge dynamics, extracted by interpolating experimental data, are reported in Supplementary Table 1.

Supplementary Note 9 | Influence of network density and topology on electrical properties.

Electrical properties of NW networks in the pristine state (i.e., before stimulation) are related to the NW density. As detailed by Forrò et al.,[18] a non-linear increase of conductivity can be observed by progressively increasing the NW density. The dependence of the conductivity of network areas on the local density of NWs was experimentally proved in our previous work by correlating local electrical properties to the local morphology of the network through combined electrical mapping, optical microscopy and transmittance spectra characterization.[15] Despite self-assembly of NW networks rely on random distribution of NWs, it is not possible to exclude that also preferential orientation of NWs in specific areas can influence the local NW network conductivity.[31]

Supplementary Note 10 | Improvement of spatial and temporal resolution of ERT mapping by optimization of measurement protocols and reconstruction algorithms.

Increasing the spatial resolution of ERT mapping relies on acquiring an increased amount of information via boundary measurements to be passed to the reconstruction algorithm. While in principle an increased amount of information can be achieved by increasing the number of electrical terminals contacting the NW network, a different approach can be explored by acquiring complementary information on the sample. To increase the number of independent measurements, one possibility is the implementation of rotational electrical impedance/resistance tomography (R-EIT).[32] In this case, the number of independent measurements is increased by moving contacts from a starting position to another distinct position on the sample boundary through the appropriate development of a rotational fixture driven by a motor, allowing to increase the overall mapping quality. However, the main drawback of this approach is the introduction of positioning uncertainty related to the mechanical movement. An example on how it is possible to increase the amount of information on the sample by acquiring complementary information was demonstrated by coupling boundary electrical measurements with magnetic measurements through magnetic resonance electrical impedance tomography (MREIT).[33] Here, an MRI scanner was exploited to provide additional independent information on the sample conductivity by measuring the induced internal magnetic flux density produced by injected currents and electric fields inside the sample, allowing to obtain higher-resolution conductivity images. All these approaches can be clearly envisaged to increase the spatial resolution of NW network mapping.

Increasing the temporal resolution of ERT mapping relies on exploiting fastest strategies for acquiring the impedance matrix of the multiterminal system. Within the context of an adjacent scanning strategy (used in the present work) and 16-contacts, data acquisition speed of well beyond 1k frames per seconds (fps) can be achieved as shown in other fields of application.[34,35] More recent approaches based on AC-regime excitation and parallel sensing of all the contacts have been proposed to reach

about 800 frames per second image reconstruction rate with real time reconstruction.[36] In this context, the measurement speed of the set of electrical measurements required for ERT reconstruction of NW networks can be increased by exploring measurement protocols in the AC regime.

References

1. He, Y., Su, X., Xu, M. & Wang, H. Analysis of electrical tomography sensitive field based on multi-terminal network and electric field. in 75441Y (2010). doi:10.1117/12.885608.
2. Cultrera, A. *et al.* Mapping the conductivity of graphene with Electrical Resistance Tomography. *Sci Rep* **9**, 10655 (2019).
3. Li, Q. *et al.* Dynamic Electrical Pathway Tuning in Neuromorphic Nanowire Networks. *Adv Funct Mater* **30**, 2003679 (2020).
4. Manning, H. G. *et al.* Emergence of winner-takes-all connectivity paths in random nanowire networks. *Nat Commun* **9**, 3219 (2018).
5. Nirmalraj, P. N. *et al.* Manipulating Connectivity and Electrical Conductivity in Metallic Nanowire Networks. *Nano Lett* **12**, 5966–5971 (2012).
6. Peter R., H. Synaptic density in human frontal cortex — Developmental changes and effects of aging. *Brain Res* **163**, 195–205 (1979).
7. Glover, G. H. Overview of functional magnetic resonance imaging. *Neurosurgery Clinics of North America* vol. 22 133–139 Preprint at https://doi.org/10.1016/j.nec.2010.11.001 (2011).
8. Milano, G., Miranda, E. & Ricciardi, C. Connectome of memristive nanowire networks through graph theory. *Neural Networks* **150**, 137–148 (2022).
9. Heitz, J., Leroy, Y., Hébrard, L. & Lallement, C. Theoretical characterization of the topology of connected carbon nanotubes in random networks. *Nanotechnology* **22**, 345703 (2011).
10. Milano, G. *et al.* Brain-Inspired Structural Plasticity through Reweighting and Rewiring in Multi-Terminal Self-Organizing Memristive Nanowire Networks. *Advanced Intelligent Systems* **2**, 2000096 (2020).
11. Milano, G. *et al.* Self-limited single nanowire systems combining all-in-one memristive and neuromorphic functionalities. *Nat Commun* **9**, 5151 (2018).
12. Wang, Z. *et al.* Memristors with diffusive dynamics as synaptic emulators for neuromorphic computing. *Nat Mater* **16**, 101–108 (2017).
13. Wang, W. *et al.* Surface diffusion-limited lifetime of silver and copper nanofilaments in resistive switching devices. *Nat Commun* **10**, 81 (2019).
14. Cultrera, A. & Callegaro, L. Electrical Resistance Tomography of Conductive Thin Films. *IEEE Trans Instrum Meas* **65**, 2101–2107 (2016).
15. Milano, G. *et al.* Mapping Time-Dependent Conductivity of Metallic Nanowire Networks by Electrical Resistance Tomography toward Transparent Conductive Materials. *ACS Appl Nano Mater* acsanm.0c02204 (2020) doi:10.1021/acsanm.0c02204.
16. Cultrera, A. *et al.* Recommended implementation of electrical resistance tomography for conductivity mapping of metallic nanowire networks using voltage excitation. *Sci Rep* **11**, 13167 (2021).

17. Montano, K., Milano, G. & Ricciardi, C. Grid-graph modeling of emergent neuromorphic dynamics and heterosynaptic plasticity in memristive nanonetworks. *Neuromorphic Computing and Engineering* 0–22 (2022) doi:10.1088/2634-4386/ac4d86.

18. Forró, C., Demkó, L., Weydert, S., Vörös, J. & Tybrandt, K. Predictive Model for the Electrical Transport within Nanowire Networks. *ACS Nano* **12**, 11080–11087 (2018).

19. Sannicolo, T. *et al.* Electrical Mapping of Silver Nanowire Networks: A Versatile Tool for Imaging Network Homogeneity and Degradation Dynamics during Failure. *ACS Nano* **12**, 4648–4659 (2018).

20. Venezian, G. On the resistance between two points on a grid. *Am J Phys* **62**, 1000–1004 (1994).

21. Nedaaee Oskoee, E. & Sahimi, M. Electric currents in networks of interconnected memristors. *Phys Rev E* **83**, 031105 (2011).

22. Li, Q., Khiat, A., Salaoru, I., Xu, H. & Prodromakis, T. Origin of stochastic resistive switching in devices with phenomenologically identical initial states. *Proceedings - IEEE International Symposium on Circuits and Systems* 1428–1431 (2014) doi:10.1109/ISCAS.2014.6865413.

23. Caravelli, F. *et al.* Mean Field Theory of Self-Organizing Memristive Connectomes. (2023) doi:10.1002/andp.202300090.

24. Milano, G. *et al.* In materia reservoir computing with a fully memristive architecture based on self-organizing nanowire networks. *Nat Mater* **21**, 195–202 (2022).

25. Milano, G., Montano, K. & Ricciardi, C. In materia implementation strategies of physical reservoir computing with memristive nanonetworks. *J Phys D Appl Phys* **56**, 084005 (2023).

26. Adler, A. *et al.* GREIT: a unified approach to 2D linear EIT reconstruction of lung images. *Physiol Meas* **30**, S35–S55 (2009).

27. Cultrera, A. & Callegaro, L. Accuracy in Electrical Resistance Tomography: from measurements to maps. in *itENBIS and INRIM Joint Workshop on Mathematical and Statistical Methods for Metrology, book of abstracts* 69 (2019).

28. Buttiker, M. Symmetry of electrical conduction. *IBM J Res Dev* **32**, 317–334 (1988).

29. Chang, T., Jo, S. H. & Lu, W. Short-term memory to long-term memory transition in a nanoscale memristor. *ACS Nano* **5**, 7669–7676 (2011).

30. Miranda, E., Milano, G. & Ricciardi, C. Modeling of Short-Term Synaptic Plasticity Effects in ZnO Nanowire-Based Memristors Using a Potentiation-Depression Rate Balance Equation. *IEEE Trans Nanotechnol* **19**, 609–612 (2020).

31. Jagota, M. & Tansu, N. Conductivity of nanowire arrays under random and ordered orientation configurations. *Sci Rep* **5**, (2015).

32. Huang, C.-N., Yu, F.-M. & Chung, H.-Y. Rotational electrical impedance tomography. *Meas Sci Technol* **18**, 2958–2966 (2007).

33. Woo, E. J. & Seo, J. K. Magnetic resonance electrical impedance tomography (MREIT) for high-resolution conductivity imaging. *Physiol Meas* **29**, R1–R26 (2008).

34. Mi Wang *et al.* A high-performance EIT system. *IEEE Sens J* **5**, 289–299 (2005).

35. Wilkinson, A. J. *et al.* A 1000-measurement frames/second ERT data capture system with real-time visualization. *IEEE Sens J* **5**, 300–307 (2005).

36. Dupre, A., Ricciardi, G. & Bourennane, S. Novel Approach for Analysis and Design of High-Speed Electrical Impedance Tomographic System for Void Fraction Measurements in Fast Two-Phase Flows. *IEEE Sens J* **17**, 4472–4482 (2017).
