# Supplementary material for: Tomography of memory engrams in self-organizing nanowire connectomes
Source: Nat Commun. 2023 Sep 27;14:5723. doi: 10.1038/s41467-023-40939-x (PMC10533552; doi:10.1038/s41467-023-40939-x)
Supplement: Supplementary file 3 — Description of Additional Supplementary Files [file 41467_2023_40939_MOESM3_ESM.docx]

**Tomography of memory engrams in self-organizing nanowire connectomes – Description of Additional Supplementary Files**

Gianluca Milano^1*^, Alessandro Cultrera^2^, Luca Boarino^1^, Luca Callegaro^2^, Carlo Ricciardi^3*^

^1^Advanced Materials Metrology and Life Sciences Division, INRiM (Istituto Nazionale di Ricerca Metrologica), Strada delle Cacce 91, 10135 Torino, Italy.

^2^Quantum Metrology and Nanotechnologies Division, INRiM (Istituto Nazionale di Ricerca Metrologica), Strada delle Cacce 91, 10135 Torino, Italy.

^3^Department of Applied Science and Technology, Politecnico di Torino, C.so Duca degli Abruzzi 24, 10129 Torino, Italy.

*e-mails: [g.milano@inrim.it](mailto:g.milano@inrim.it); [carlo.ricciardi@polito.it](mailto:carlo.ricciardi@polito.it);

**File:** Supplementary Movie 1

**Description:** Animation of the experimental dynamic evolution of memory traces in a homogeneous memristive NW connectome. The animation, that refers to experimental data reported in Figure 3, shows the dynamic evolution of the effective conductance, of the differential impedance matrices and the corresponding differential conductivity maps after stimulation of the network in between the selected pair of neuron terminals (6,15) with a 3V voltage pulse of 10s. The animation shows the emergence of an activation pattern with enhanced conductivity connecting the stimulated terminals that progressively vanish over time after the end of stimulation due to the short-term memory characteristic of the network connectome.

**File:** Supplementary Movie 2

**Description:** Animation of the dynamic evolution of memory traces in a memristive NW network simulated through grid-graph modeling. The animation, that refers to grid-graph modeling data reported in Figure 3, shows the dynamic evolution of the modelled effective conductance, of the grid-graph model (red intensity is proportional to the edge conductance, blue intensity is proportional to the node voltage), of the effective conductance, of the differential impedance matrices obtained through the grid-graph model and the corresponding differential conductivity maps during and after stimulation of the network in between the selected pair of neuron terminals (6,15) with a 3V voltage pulse of 10s. Grid-graph model parameters reported Supplementary Table 1 were retrieved from experimental data. The animation shows the gradual formation of a conductive pathway with enhanced conductivity growing over time starting from the stimulated electrodes along the electric field gradient during stimulation and its subsequent progressive vanishing due to the short-term memory of the network. The dynamic evolution of the synaptic pathway is in qualitative agreement with experimental results obtained on nearly homogenous NW networks (refer to Supplementary Video 1).

**File:** Supplementary Movie 3

**Description:** Animation of the experimental dynamic evolution of memory traces in a non-homogeneous memristive NW network with a relatively low amplitude stimulation showing short-term memory effects. The animation, that refers to experimental data reported in Figure 4, shows the dynamic evolution of the effective conductance, of the differential impedance matrices and the corresponding differential conductivity maps after stimulation of the network in between the selected pair of neuron terminals (6,13) with a 1V voltage pulse of 10s. The animation shows the emergence of enhanced conductivity areas near stimulating terminals, with a larger stimulated area near terminal 6 respect to terminal 13. The asymmetry of the spatial activation pattern is inherently related to the network topology and the pristine state conductivity map (Figure 4a). Activation patterns tends to vanish over time as a consequence of short-term memory effects of the network.

**File:** Supplementary Movie 4

**Description:** Animation of the experimental dynamic evolution of memory traces in a non-homogeneous memristive NW network connectome with a relatively high amplitude stimulation showing long-lasting memory effects. The animation, that refers to experimental data reported in Figure 4, shows the dynamic evolution of the effective conductance, of the differential impedance matrices and the corresponding differential conductivity maps after stimulation of the network in between the selected pair of neuron terminals (6,13) with a 2V voltage pulse of 10s. The animation shows the emergence of a conductive area connecting stimulating terminals with enhanced conductivity respect to the activation pattern generated by stimulation with a lower amplitude (refer to Supplementary Movie 3). In this case, long-lasting changes in impedance matrices reflect in activation patterns across the network that tend to be stable over time, showing long-lasting changes in the network connectome.
